# Supplementary material for: Model-Based Geostatistical Mapping of the Prevalence of Onchocerca volvulus in Cameroon between 1971 and 2020
Source: PLoS Negl Trop Dis. 2025 Mar 28;19(3):e0012250. doi: 10.1371/journal.pntd.0012250 (PMC11978065; doi:10.1371/journal.pntd.0012250)
Supplement: S1 File — Table A. Definition of the bioclimatic variables used. Text A. Conversion of nodule prevalence into microfilarial prevalence. Text B. Description of the Model-Based Geostatistical (MBG) approach used and parameter estimation procedures. Table B. Parameter estimates and 95% confidence limits (CL) for the binomial geostatistical model of the association between microfilarial prevalence and environmental covariates for the baseline (1971–2000) period, and spatial covariance parameters. Table C. Parameter estimates and 95% confidence limits (CL) for the binomial geostatistical model of the association between microfilarial prevalence and environmental covariates for the 2001–2010 period, and spatial covariance parameters. Table D. Parameter estimates and 95% confidence limits (CL) for the binomial geostatistical model of the association between microfilarial prevalence and environmental covariates for the 2011–2020 period, and spatial covariance parameters. Text C. Interpretation of environmental coefficients. (PDF) [file pntd.0012250.s001.pdf]

Supporting Information S1 File. Detailed methods

Model-Based Geostatistical Mapping of the Prevalence of *Onchocerca volvulus* in Cameroon between 1971 and 2020

Yannick Niamsi-Emalio[1,2,*], Hugues C. Nana-Djeunga[1], Claudio Fronterrè[3], Himal Shrestha[4], Georges B. Nko'Ayissi[5], Théophile M. Mpaba Minkat[6], Joseph Kamgno[1,2], María-Gloria Basáñez[7,*]

[1] Higher Institute for Scientific and Medical Research, Yaoundé, Cameroon

[2] Department of Public Health, Faculty of Medicine and Biomedical Sciences, University of Yaoundé I, Yaoundé, Cameroon

[3] Centre for Health Informatics, Computing and Statistics, Lancaster Medical School, Lancaster University, Lancaster, United Kingdom

[4] Department of Microbiology and Immunology, University of Melbourne at the Peter Doherty Institute for Infection and Immunity, Melbourne, Victoria, Australia

[5] Neglected Tropical Diseases National Coordination Unit, Ministry of Public Health, Yaoundé, Cameroon

[6] National Onchocerciasis Control Programme, Ministry of Public Health, Yaoundé, Cameroon

[7] MRC Centre for Global Infectious Disease Analysis and London Centre for Neglected Tropical Disease Research, School of Public Health, Imperial College London, London, United Kingdom.

*Correspondence:

emalio2002@yahoo.fr (ORCID: 0000-0002-5766-6255); m.basanez@imperial.ac.uk (ORCID: 0000-0001-5031-3361)

Table A. Definition of the bioclimatic variables used [1].

Label	Definition
BIO1	Annual Mean Temperature
BIO2	Mean Diurnal Range (Mean of monthly (max temp - min temp))
BIO3	Isothermality (BIO2/BIO7) (×100)
BIO4	Temperature Seasonality (standard deviation ×100)
BIO5	Max Temperature of Warmest Month
BIO6	Min Temperature of Coldest Month
BIO7	Temperature Annual Range (BIO5 – BIO6)
BIO8	Mean Temperature of Wettest Quarter
BIO9	Mean Temperature of Driest Quarter
BIO10	Mean Temperature of Warmest Quarter
BIO11	Mean Temperature of Coldest Quarter
BIO12	Annual Precipitation
BIO13	Precipitation of Wettest Month
BIO14	Precipitation of Driest Month
BIO15	Precipitation Seasonality (Coefficient of Variation)
BIO16	Precipitation of Wettest Quarter
BIO17	Precipitation of Driest Quarter
BIO18	Precipitation of Warmest Quarter
BIO19	Precipitation of Coldest Quarter

Text A. Conversion of nodule prevalence into microfilarial prevalence

We used the procedure described by Coffeng (2024) in the Zenodo repository https://zenodo.org/records/13969100 [2].

This repository provides a set of posterior distribution draws (posterior_sample.csv) and instructions for their use (posterior_sample_instructions.docx) to convert onchocercal nodule prevalence in adult males (aged ≥20 years) into microfilarial prevalence in the general population (aged ≥5 years). The posterior draws are based on the analysis of (paired) field data on prevalence of nodules and skin microfilariae from onchocerciasis-endemic villages presented in Coffeng et al. (2013) [3], and in particular on the detailed description of the statistical model given in Supplementary S1 Text [3]. Briefly, the conditional distribution of

village-level microfilarial prevalence given nodule prevalence is formulated using (univariate or multivariate) normal distributions for the logit-transformed prevalences (parameterised in terms of mean and variance or covariance). The observed, ‘apparent’ nodule prevalence is corrected according to the diagnostic performance parameters of nodule palpation to provide ‘true’ nodule prevalence. The comma-separated ”posterior_sample.csv” file given in [2] contains a large sample of draws from the joint posterior distribution of the vector of overall mean microfilarial prevalence and nodule prevalence and its covariance, village-level standard deviation of microfilarial and nodule prevalence and their correlation, and specificity of nodule palpation. An algorithm is provided in the “posterior_sample_instructions.docx” file to generate a posterior predictive draw for microfilarial prevalence, conditional on a posterior draw of nodule prevalence [2].

Text B. Description of the Model-Based Geostatistical (MBG) approach used and parameter estimation procedures

Methodological framework

To model the spatial variation of *Onchocerca volvulus* microfilarial prevalence, a MBG approach was used to take into account the spatial correlation between observations and the effects of environmental covariates following Diggle & Giorgi [4] and Giorgi et al. [5]. We followed the procedures described in Diggle & Giorgi [4] and O’Hanlon et al. [6].

Let Y_i be the number of onchocerciasis-positive individuals in an examined population sample of size m_i living at locality x_i. If $p(x_i)$ designates the *O. volvulus* microfilarial prevalence at locality x_i, (i.e. the probability of being onchocerciasis-positive at locality x_i), the distribution of Y_i follows a binomial distribution with parameters m_i and p_i, $Y_i \sim Bin(m_i, p_i)$. Thus, conditional on an unknown stochastic Gaussian spatial process $S(\cdot)$, i.e., the unobserved, underlying spatial process, a logistic regression mixed-effects model can be formulated to account for extra-binomial variation,

$$\text{Log}\left(\frac{p(x_i)}{1-p(x_i)}\right) = T_i = \sum_{j=1}^{k} d_j(x_i)^{\mathsf{T}}\beta_j \; + S(x_i) + Z_i \qquad \text{(S1)}$$

where $d_j(x_i)^{\mathsf{T}}$ represents a vector of environmental covariates (the jth environmental covariate at locality x_i); β_j the regression coefficient of the jth variable; $S(x_i)$ the spatially correlated random effect, and Z_i a random effect deriving from a standard mixed-effects model [4,6]. Z_i follows a centered normal distribution with variance τ^2, i.e., $Z_i \sim N(0, \tau^2)$; $S(\cdot) = \{S(x) : x \in \mathbb{R}^2\}$ is a Gaussian process with mean zero, variance σ^2 and correlation function $\rho(x, x') = \text{Corr}\big(S(x), S(x')\big) = \rho\|x - x'\| = \rho(u)$, u being the Euclidian distance between two sampling locations, x and x' [4]. The Matérn correlation function was used, which is given by,

$$\rho(u;\, \phi, \kappa) = (2^{\kappa-1}\Gamma(\kappa))^{-1}(u/\phi)^{\kappa}\kappa_{\kappa}(u/\phi), u > 0 \qquad \text{(S2)}$$

where $\phi > 0$ is the scale (or range) parameter, $\Gamma(\cdot)$ is the gamma function, and $\kappa_{\kappa}(\cdot)$ is the modified Bessel function of order $\kappa > 0$.

The parameters of the model, β and $\theta^{\mathsf{T}} = (\,\sigma^2, \phi, \tau^2)$, were estimated using Monte Carlo maximum likelihood (MCML) [7,8]. Here, σ^2 is the 'sill' or maximum variance of the spatial process and τ^2 is the nugget variance, i.e., the non-spatial variance (representing sources of non-spatial variation) [6]. The nugget variance, τ^2—not a parameter estimated by the model—was specified as a relative nugget (i.e. as the proportion of the total variance of the spatial process that is due to non-spatial effects). The relative nugget was computed using the parameter estimates from the theoretical variogram model fitting procedure [6] (see *Initializing the model* sub-section below). The MCML method uses conditional simulation of the distribution of T given Y to approximate the multidimensional integral of the model likelihood given by Eqn. (S4) below.

Monte Carlo maximum likelihood

The likelihood function for the parameters β and $\theta^{\mathsf{T}} = (\,\sigma^2, \phi, \tau^2)$, is obtained by integrating out the random effects in T_i as defined by Eqn. (S1). Let *D* be the matrix with *n* rows and *k* columns

corresponding to the k explanatory variables of the model and $y^{\top} = (y_1,\cdots, y_n)$ the vector of onchocerciasis-positive cases (binomial observations). The marginal distribution of T is multivariate Gaussian with mean vector $D\beta$ and variance–covariance matrix $\Sigma(\theta)$ consisting of values $\sigma^2 + \tau^2$ on the main diagonal and values $\sigma^2\rho(u_{ij})$ off-diagonal, where u_{ij} is the distance between localities x_i and x_j. The conditional distribution of $Y^{\top} = (Y_1, \ldots, Y_n)$ given $T^{\top} = (t_1,\cdots, t_n)$ is the product of the independent binomial probability functions, given by Eqn. (S3),

$$f(y|t) = \prod_{i=1}^{n} f(y_i|t_i) \tag{S3}$$

The likelihood function is defined by Eqn. (S4),

$$L(\beta, \theta) = f(y; \beta, \theta) = \int_{\mathbb{R}^n} N(t; D\beta, \Sigma(\theta))f(y|t)dt \tag{S4}$$

where $N(t; D\beta, \Sigma(\theta))$ is a multivariate Gaussian distribution with mean $D\beta$ and variance–covariance matrix $\Sigma(\theta)$. To perform conditional simulations of T given $Y = y$ with parameters β and θ, the MCML method uses the Langevin-Hastings algorithm. To implement the model defined by Eqn. (S1), the *binomial.logistic.MCML* function of the PrevMAP package version 1.5.4 was used [9].

Initializing the model

Executing the *binomial.logistic.MCML* function requires initializing parameters β and θ. The initial values θ_0 of θ were obtained by modelling the semi-variograms of the data for the study periods (1971–2000, 2001–2010; 2011–2020) and estimating the values $(\sigma_0^2, \phi_0, \tau_0^2)$. The Matérn correlation function (Eqn. (S2)) was used. To obtain initial values of β, generalized linear regression (GLM) models for binary dependent variables (logistic regressions without the spatial component), were implemented for both estimating β_0 and selecting the environmental covariates β_j to be included into the model of Eqn. (S1). For each GLM, and using a step-wise approach based on minimizing the Akaike Information Criterion (AIC), only

statistically significant variables were kept (with significance set at 0.05). Multicollinearity checks were carried out using analysis of variance inflation factors (VIF) [10,11],

$$VIF_j = \frac{1}{1 - R_j^2} \tag{S5}$$

where R_j^2 is the coefficient of determination of the jth predictor when the latter is explained by the other predictor variables [10]. Predictors with VIF greater than 10 were removed from the model using a backward procedure. The GLM models retained contained only significant predictors whose VIFs were less than 10. The predictors retained were used to implement Eqn. (S1) and the coefficients β_0 estimated were used for the initialization of the MCML method. The MCML method was run three times for each study period to ensure convergence and stability of parameter estimates β and θ. All analyses were performed with R version 4.4.2 [12].

Spatial prediction

Prediction of $T^* = \left(T(x_{n+1}), \ldots, T(x_{n+q})\right)^{\mathsf{T}}$ at q additional unsampled locations requires all relevant explanatory variables to be available at the prediction locations. We do not include Z_i of Eqn (S1) as part of our prediction target. If data are available at $x_i, i = 1 \ldots n$ locations, and predictions made at $x_{n+i}, i = 1 \ldots q$,

$$T_{(x_{n+i})} = \sum_{j=1}^{k} d_j(x_{n+i})^{\mathsf{T}} \beta_j + S(x_{n+i}) \tag{S6}$$

Conditionally on $T^{\mathsf{T}} = (T_1, \cdots, T_n)$, β, θ, and y, the target for prediction T^*follows a multivariate Gaussian distribution with mean $\mu^*(T) = D^*\beta + C\Sigma^{-1}(T - D\beta)$ and covariance matrix $\Sigma^* = V - C\Sigma^{-1}C^{\mathsf{T}}$, where C is the cross-covariance matrix between T and T^*, V is the covariance matrix of T^*, and $D^*$is a q by k matrix of explanatory variables at prediction locations [9].

Exceedance probability

Of particular interest in problems of hotspot detection, is the exceedance probability $P(T(x_{n+i}) > l|y)$ for a given threshold l and $i = 1, \dots q$,

$$P(T(x_{n+i}) > l|y) = \frac{1}{m} \sum_{j=1}^{m} I\big(T_{(j)}(x_{n+1}) > l\big) \tag{S7}$$

where $I(a > l)$ is 1 if $a > l$ and 0 otherwise, and $T_{(j)}(x_{n+i})$ is the i-th element of $T_{(j)}^{*}$ [9].

Table B. Parameter estimates and 95% confidence limits (CL) for the binomial geostatistical model of the association between microfilarial prevalence and environmental covariates for the baseline (1971–2000) period, and spatial covariance parameters.

Variable	Coefficient	Lower 95% CL	Upper 95% CL	p-value
Intercept	5.966	-4.822	16.754	0.2784
NDVI[1] (1st quarter: Dec–Feb)	0.422	-2.197	3.041	0.7521
NDVI[1] (4th quarter: Sep–Nov)	0.204	-3.822	4.230	0.9209
BIO3: Isothermality	-0.129	-0.239	-0.019	0.0218
BIO4: Temperature seasonality	-0.013	-0.031	0.005	0.1533
BIO9: Mean temperature of driest quarter	0.181	0.009	0.353	0.0396
BIO13: Precipitation of wettest month	-0.002	-0.010	0.006	0.5994
BIO14: Precipitation of driest month	-0.016	-0.061	0.029	0.4756
$\boldsymbol{\phi}$, range parameter (Km)	98.866	98.858	98.874	–
$\boldsymbol{\sigma^2}$, Sill	2.402	2.171	2.633	–

[1]NDVI: Normalised difference vegetation index.

Table C. Parameter estimates and 95% confidence limits (CL) for the binomial geostatistical model of the association between microfilarial prevalence and environmental covariates for the 2001–2010 period, and spatial covariance parameters.

Variable	Coefficient	Lower 95% CL	Upper 95% CL	p-value
Intercept	1.574	-3.457	6.605	0.5397
NDVI[1] (3rd quarter Jun–Aug)	-1.606	-5.465	2.253	0.4148
Elevation	0.000	-0.002	0.002	0.4361
BIO4: Temperature seasonality	-0.008	-0.020	0.004	0.1933
BIO13: Precipitation of wettest month	-0.007	-0.017	0.003	0.1819
BIO14: Precipitation of driest month	-0.027	-0.080	0.026	0.3297
BIO18: Precipitation of warmest quarter	0.003	-0.001	0.007	0.2537
BIO19: Precipitation of coldest quarter	0.001	-0.001	0.003	0.2857
$\boldsymbol{\phi}$, range parameter (Km)	43.728	43.716	43.740	–
$\boldsymbol{\sigma^2}$, Sill	1.874	1.668	2.080	–

[1]NDVI: Normalised difference vegetation index.

Table D. Parameter estimates and 95% confidence limits (CL) for the binomial geostatistical model of the association between microfilarial prevalence and environmental covariates for the 2011–2020 period, and spatial covariance parameters.

Variable	Coefficient	Lower 95% CL	Upper 95% CL	p-value
Intercept	-5.322	-25.620	14.976	0.6073
NDVI[1] (1st quarter: Dec–Feb)	2.946	-3.234	9.126	0.3502
NDVI[1] (2nd quarter: Mar–May)	-3.127	-10.767	4.513	0.4225
NDVI[1] (4th quarter: Sep–Nov)	-2.108	-9.497	5.281	0.576
BIO3: Isothermality	0.045	-0.137	0.227	0.6289
BIO4: Temperature seasonality	0.024	-0.007	0.055	0.1434
BIO5: Max temperature of warmest month	-0.228	-0.634	0.178	0.2703
BIO6: Min temperature of coldest month	0.443	0.018	0.868	0.0414
BIO13: Precipitation of wettest month	-0.011	-0.021	-0.001	0.0157
BIO14: Precipitation of driest month	0.005	-0.052	0.062	0.8722
BIO18: Precipitation of warmest quarter	0.005	-0.001	0.011	0.1529
$\boldsymbol{\phi}$, range parameter (Km)	56.031	56.017	56.045	–
$\boldsymbol{\sigma^2}$, Sill	2.432	2.211	2.653	–

[1]NDVI: Normalised difference vegetation index.

Text C. Interpretation of environmental coefficients

For the model fitted to the entire study interval (1971–2020, Table 1 of the main text), the normalised difference vegetation index (NDVI) of the 3rd quarter (Jun–Aug) was statistically significant and negatively associated with prevalence. This quarter corresponds to a dry season period in Cameroon, which leads to a reduction in the volume of watercourses necessary for blackfly reproduction. In the 2001–2010 period, none of the environmental covariates were statistically significant (Table C), suggesting that all the variability in

prevalence was explained by the spatial correlation. For 2011–2020, BIO6 (minimum temperature of the coldest month) and BIO13 (precipitation of the wettest month) were, respectively, positively and negatively significantly associated with prevalence (Table D), the former presumably owing to conditions favouring development of blackfly immature stages, parasite larval development within the vectors and vector survival, and the latter due to detrimental impacts of increased precipitation on vector breeding sites [13].

Supplementary references

1. WorldClim. Bioclimatic variables. 2020–2022. Available from: https://www.worldclim.org/data/bioclim.html
2. Coffeng LE. Onchocerciasis: the pre-control association between prevalence of palpable nodules and skin microfilariae - technical note and posterior draws for conversion equation. 2024. Available from: https://zenodo.org/records/13969100. https://doi.org/10.5281/zenodo.13969100.
3. Coffeng LE, Pion SDS, O'Hanlon S, Cousens S, Abiose AO, Fischer PU, et al. Onchocerciasis: the pre-control association between prevalence of palpable nodules and skin microfilariae. PLoS Negl Trop Dis. 2013;7: e2168. https://doi.org/10.1371/journal.pntd.0002168.
4. Diggle PJ, Giorgi E. Model-based geostatistics for prevalence mapping in low-resource settings. J Am Stat Assoc. 2016;111: 1096–1120. https://doi.org/10.1080/01621459.2015.1123158.
5. Giorgi E, Fronterrè C, Macharia PM, Alegana VA, Snow RW, Diggle PJ. Model building and assessment of the impact of covariates for disease prevalence mapping in low-resource settings: to explain and to predict. J R Soc Interface. 2021;18: 20210104. https://doi.org/10.1098/rsif.2021.0104.
6. O'Hanlon SJ, Slater HC, Cheke RA, Boatin BA, Coffeng LE, Pion SDS, Boussinesq M, Zouré HGM, Stolk WA, Basáñez MG. Model-based geostatistical mapping of the

prevalence of *Onchocerca volvulus* in West Africa. PLoS Negl Trop Dis 2016;10: e0004328. https://doi.org/10.1371/journal.pntd.0004328.

7. Geyer CJ. Chapter 3: Likelihood inference for spatial point processes. In: Barndroff-Nielsen OE, Kendall WS, van Lieshout MNM, editors. Stochastic geometry. Likelihood and computation (1st edition). Monographs on Statistics and Applied Probability 80. London: Chapman and Hall/CRC. 1998; pp. 79–140. Available from: https://www.taylorfrancis.com/chapters/edit/10.1201/9780203738276-3/likelihood-inference-spatial-point-processes-geyer.
8. Geyer CJ, Thompson EA. Constrained Monte Carlo maximum likelihood for dependent data. J R Stat Soc Ser B (Methodol) 1992;54: 657–99. https://doi.org/10.1111/j.2517-6161.1992.tb01443.x.
9. Giorgi E, Diggle PJ. PrevMap: An R package for prevalence mapping. J Stat Softw 2017;78: 1–29. Available from: https://doi.org/10.18637/jss.v078.i08.
10. Hsieh FY, Lavori PW, Cohen HJ, Feussner JR. An overview of variance inflation factors for sample-size calculation. Eval Health Prof 2003;26: 239–57. https://doi.org/10.1177/0163278703255230.
11. Marcoulides KM, Raykov T. Evaluation of variance inflation factors in regression models using latent variable modeling methods. Educ Psychol Meas. 2019;79: 874–82. https://doi.org/10.1177/0013164418817803.
12. The R Project for Statistical Computing. Vienna, Austria. Available from: https://www.r-project.org/.
13. Cheke RA, Basáñez MG, Perry M, White MT, Garms R, Obuobie E, et al. Potential effects of warmer worms and vectors on onchocerciasis transmission in West Africa. Philos Trans R Soc Lond B Biol Sci. 2015;370: 20130559. https://doi.org/10.1098/rstb.2013.0559.
