# Supplementary material for: Model-Based Geostatistical Mapping of the Prevalence of Onchocerca volvulus in Cameroon between 1971 and 2020
Source: PLoS Negl Trop Dis. 2025 Mar 28;19(3):e0012250. doi: 10.1371/journal.pntd.0012250 (PMC11978065; doi:10.1371/journal.pntd.0012250)
Supplement: Attachment — Submitted filename: Response to Reviewers_22.12.2024.pdf [file pntd.0012250.s004.pdf]

Response to Reviewers: PNTD-D-24-00734

Model-Based Geostatistical mapping of the prevalence of *Onchocerca volvulus* in Cameroon between 1971 and 2020

Reviewer 1

1.1 General comment: This is a well-written manuscript and an important piece of work that provides geostatistical modeling predictions of onchocerciasis prevalence in Cameroon from 1971 to 2020. It offers further evidence of the effectiveness of ivermectin in controlling and eliminating onchocerciasis. The data output from this work has the potential to be applied to transmission models to inform future trends and predictions toward onchocerciasis elimination.

Reply by Authors to 1.1: We thank the referee for these appreciative comments on our manuscript.

1.2 Abstract: Well-written. It is clear, concise, and contains all the relevant details.

Reply by Authors to 1.2: We are grateful to the reviewer for finding our abstract clear, concise and relevant.

1.3 Introduction: Well-written. It provides a good historical background on interventions against onchocerciasis. However, since this work focuses on building geostatistical models for onchocerciasis in Cameroon, and not on the transmission modeling use case of geostatistical modeling outputs (Lines 172-175), it is recommended to exclude the latter from the introduction. Use cases for geostatistical modeling outputs can be provided in the Discussion section, as they are out of place in the introduction.

Reply by Authors to 1.3: We appreciate the comments by the referee. However, as the ultimate motivation of the work is to be able to link the geostatistical modelling with transmission dynamics, we prefer to include this motivation in the Introduction to provide the readers with a general direction of the work, but we have revised the text to make it more concise. We now say:

"In this paper we present a geostatistical model fitted to *O. volvulus* microfilarial prevalence across space and time in Cameroon to visualize the evolution of onchocerciasis control efforts carried out by the NOCP. The ultimate aim will be to combine MBG and transmission dynamics modelling to identify areas of the country that may reach EOT with the current CDTI approach and those that will necessitate alternative and/or complementary strategies towards the 2030 time-horizon and beyond."

1.4 Data: More information is needed in this section to understand what onchocerciasis data was used to fit the models. For example, please provide the numbers and distribution of data across all data sources. How much of the data was obtained from the literature, the ESPEN portal, etc.? For what years? Were there any data duplicates? For instance, did ESPEN's report of prevalence rates for a specific area differ from rates reported for a similar area in data obtained from the literature? If there are examples of such instances, how did the authors handle them? I see that this sections links to a protocol cited in ref 39, but it is good to provide adequate information, so readers don't have to go elsewhere to get a complete understanding of the study.

Reply by Authors to 1.4: We agree with the referee that more detail on the data that were used would be useful. In the revised version of the manuscript we provide more information on these data as follows:

"To identify data duplicates, records were compared according to region, health district, village, period, and number of people surveyed. After duplicates were removed, the data comprised 983 (70%) nodule prevalence surveys and 421 (30%) microfilarial prevalence surveys, for a total of 1,404 (village-level) georeferenced surveys during the overall (1971–2020) study interval. Of the 983 nodule prevalence surveys, 842 (86%) were obtained from the ESPEN portal, and 141 (14%) from published literature. Of the 421 microfilarial prevalence surveys, 122 (29%) were from ESPEN, 204 (48%) from the literature, and 95 (23%) from unpublished surveys."

1.5 Covariates: Onchocerciasis endemicity is not driven solely by climatic factors. Not including key socio-demographic variables as covariates in this model could be considered a significant omission. The authors should justify why they have chosen not to account for any socio-demographic covariates in this work.

Reply by Authors to 1.5: We have included the following text in the Limitations section:

"We did not include socio-economic covariates in our analysis because onchocerciasis distribution and prevalence tend to be highly focal and mostly limited to rural areas proximal to fast-flowing rivers and streams suitable to vector breeding, indicating that environmental covariates take pre-eminence [34,48]."

1.6 Statistical analysis:

1.6.1 Conversion of nodule to microfilaria prevalence and its stratification. The terms pre-intervention, baseline, and pre-control (Lines 194-196) are used interchangeably. Although they all mean the same thing, it would be better to be consistent and use one term throughout the manuscript.

Reply by Authors to 1.6.1: These various terms are used interchangeably in the literature because they do refer to the same thing. Therefore, we would prefer to keep them as they will be found by readers in this fashion.

1.6.2 Geostatistical model: It is unclear if a separate model was fitted to each of the year bands and time periods described in previous sections. From the empirical semi-variograms in Fig. 3, it appears that each time period was modeled separately. Please clarify this in this section.

Reply by Authors to 1.6.2: We fitted the model firstly to all time periods (1971–2020 interval) to assess the influence of the intervention period on the results (presented in the Main Text). Second, we fitted the model to the different time periods separately, i.e., to baseline (1971–2000), 2001–2010 and 2011–2020 periods with the results for each model presented in the Supplementary File. We have clarified this in the Methods section, where we now say:

"In the first instance, the model was fitted to the overall, 1971–2020 interval to assess the effect of intervention period. Subsequently, the model was fitted separately to the baseline (1971–2000), 2001–2010 and 2011–2020 periods."

1.7 Discussion:

1.7.1 The first paragraph in the Discussion section goes into more description of the data. This may be better suited in the Methods section, as it seems out of place to start the Discussion with this information.

Reply by Authors to 1.7.1: We have re-structured the Discussion. In its first paragraph we no longer provide any additional description of data that should go in the Methods section.

1.7.2 To improve flow and readability, I would also recommend re-ordering the Discussion as follows:

- Implications of our findings for onchocerciasis elimination in Cameroon
- MBG mapping of onchocerciasis in Cameroon
- Limitations
- Future research avenues

Reply by Authors to 1.7.2: We have re-ordered the Discussion but would prefer to start with a summary of our results followed by their policy implications. We now have the following sub-headings:

- MBG mapping of onchocerciasis in Cameroon
- Implications of our findings for onchocerciasis elimination in Cameroon
- Limitations
- Future research avenues

1.8 Use of the word 'significant' – The authors should use 'statistically significant' if that is what they intend to convey.

Reply by Authors to 1.8: We have added 'statistically' to 'significant' wherever appropriate.

Reviewer 2

2.1 Summary and General Comments: This is an interesting analysis of collated onchocerciasis prevalence data and much of it is well written and clear. Areas of improvement are the following: methods (clarification needed on the modelling approach and more information needed about the data used); results (some minor edits to figures and captions required to make them more interpretable; more specific language required relating to models used and mean predicted prevalence shown in plots); discussion (requires the most work – needed to cut and focus the existing text to make it more readable and to clearly communicate your key points; there are some really interesting points in this section however!).

Reply by Authors to 2.1: We thank the referee for the appreciative comments about the interest of our manuscript. We will address the various points raised separately.

2.2 Objectives: Clear study objectives and time periods. The overview of the study population, unit of analysis and data sources used for modelling could be improved (a couple of minor comments below). Suitable MBG approach followed for the analysis though a few points could be explained more clearly about the model selection. No ethical concerns.

Reply by Authors to 2.2: We are pleased to see that the referee states that the study objectives and time periods are clear, as well as that we have used a suitable MBG approach.

2.3 Methods:

2.3.1 Data overview: I appreciate this analysis is being conducted on data from a diverse range of different studies already described in the systematic review, but it would be helpful to be given a brief overview of what kind of studies these consist of, i.e. are they all school/household/community prevalence surveys, what diagnostics were used etc. To help the reader understand the complexities of jointly modelling all of this data together, particularly if anything changes over the study periods in terms of how data was collected that could have biased the results.

Reply by Authors to 2.3.1: The surveys were either microfilarial prevalence surveys conducted at village level (not at school or household level) or nodule prevalence surveys, also conducted at village level. The latter were based on examining adult males in the village for palpable (sub-cutaneous) onchocercal nodules. We have added the following text to the revised version:

"To identify data duplicates, records were compared according to region, health district, village, period, and number of people surveyed. After duplicates were removed, the data comprised 983 (70%) nodule prevalence surveys and 421 (30%) microfilarial prevalence surveys, for a total of 1,404 (village-level) georeferenced surveys during the overall (1971–2020) study interval. Of the 983 nodule prevalence surveys, 842 (86%) were obtained from the ESPEN portal, and 141 (14%) from published literature. Of the 421 microfilarial prevalence surveys, 122 (29%) were from ESPEN, 204 (48%) from the literature, and 95 (23%) from unpublished surveys. Microfilarial prevalence surveys were conducted by the skin-snip microscopy method. For this, two skin snips (one from each iliac crest) were taken from each examined individual (aged ≥5 years) with a 2-mm Holth corneoscleral punch and incubated in saline solution to allow emergence of the microfilariae, whose presence was detected by examining the incubation medium under a microscope [37]. Nodule prevalence surveys were conducted by detecting the presence of palpable (subcutaneous) onchocercomas in adult males residing in the villages [30,32,33]."

2.3.2 Spatial resolution: I would include the spatial resolution of the environmental covariates used in the analysis.

Reply by Authors to 2.3.2: All environmental data were extracted from the NASA portal, at 5 km x 5 km resolution. We have now added this information to the Methods section:

"Environmental data, including altitude (elevation), topography, Normalized Difference Vegetation Index (NDVI), and bioclimatic variables (BIO1 to BIO19) were downloaded from the National Aeronautics and Space Administration (NASA) portal [39] at 5 km x 5 km spatial resolution. The definition of the bioclimatic variables can be found in the Supporting Information file, S1 Table."

2.3.3 Nodule prevalence conversion: It would be helpful to include in the article or supplementary material how nodule prevalence was converted into microfilarial prevalence, not just cited (lines 190-191).

Reply by Authors to 2.3.3: We have added the following in the Main Text, and have included a new section in the Supporting Information File under the new S1 Text:

"As the data collated included both microfilarial and nodule prevalence, nodule prevalence (in samples of adult males aged ≥20 years [30,32,33]) was converted into microfilarial prevalence (in those aged ≥5 years) [40] (Supporting Information file, S1 Text)."

S1 Text. Conversion of nodule prevalence into microfilarial prevalence

"We used the procedure described by Coffeng (2024) in the Zenodo repository https://zenodo.org/records/13969100 [2].

This repository provides a set of posterior distribution draws (posterior_sample.csv) and instructions for their use (posterior_sample_instructions.docx) to convert onchocercal nodule prevalence in adult males (aged ≥20 years) into microfilarial prevalence in the general population (aged ≥5 years). The posterior draws are based on the analysis of (paired) field data on prevalence of nodules and skin microfilariae from onchocerciasis-endemic villages presented in Coffeng et al. (2013) [3], and in particular on the detailed description of the statistical model given in Supplementary S1 Text [3]. Briefly, the conditional distribution of village-level microfilarial prevalence given nodule prevalence is formulated using (univariate or multivariate) normal distributions for the logit-transformed prevalences (parameterised in terms of mean and variance or covariance). The observed, 'apparent' nodule prevalence is corrected according to the diagnostic performance parameters of nodule palpation to provide 'true' nodule prevalence. The comma-separated "posterior_sample.csv" file given in [2] contains a large sample of draws from the joint posterior distribution of the vector of overall mean microfilarial prevalence and nodule prevalence and its covariance, village-level standard deviation of microfilarial and nodule prevalence and their correlation, and specificity of nodule palpation. An algorithm is provided in the "posterior_sample_instructions.docx" file to generate a posterior predictive draw for microfilarial prevalence, conditional on a posterior draw of nodule prevalence [2]."

We added new references [2] and [3] to the Supplementary references.

2.3.4 Endemicity levels: 'The microfilarial prevalence endemicity levels described in [27] were used for stratification.' (lines 196-197), please state the endemicity levels used in the analyses here.

Reply by Authors to 2.3.4: These levels had already been described in the Introduction, when Ref. [27] was first mentioned:

"In onchocerciasis, endemicity is categorized according to all-ages microfilarial prevalence into:
i) hypoendemicity (>0% – <35%), ii) mesoendemicity (≥35% – <60%) and iii) hyperendemicity (≥60%) owing to their relationship with blindness prevalence [27]."

However, and to address query **2.4.1** below (regarding the visualisations of the display items), we have now used the same microfilarial prevalence scale for Fig 1 and Fig 2. We now state in the Methods:

"For the pre-control map, the microfilarial prevalence endemicity levels described above [27] were used for stratification. However, and to better discern locations with low prevalence in both the pre-control and subsequent maps, the additional category of 0% was used for those records reporting 0% prevalence, and the >0 – <35% category was subdivided into >0% – <10% and ≥10% – <35%."

We also added the following text to the caption of Fig 1:

“The hypoendemicity level (>0% – <35% microfilarial prevalence) has been subdivided into >0% – <10% and ≥10% – <35% microfilarial prevalence to facilitate comparison with Fig 2.”

2.3.5 Data-points: Lines 200-201: ‘We identified a total of 571 georeferenced prevalence survey data-points for the 1971–2000 period; 410 for 2001– 2005; 98 for 2006–2010; 261 for 2011–2015 and 64 for 2016–2020, for a total of 1,404.’ – This isn’t clear, are you saying that there were 1,404 prevalence survey datapoints geolocated at 571 unique locations? It’s also not clear what a datapoint is here – is it an individual, a school? Please explain to the reader to give an idea of the unit of analysis and sample size.

Reply by Authors to 2.3.5: A data-point corresponds to a georeferenced prevalence survey at village level, not to an individual or a school. Onchocerciasis is not like schistosomiasis or soil-transmitted helminthiases, in which surveys conducted only in school-aged children may give a reasonable representation of the epidemiological situation. For each period, a number of distinct prevalence surveys was identified, but not all villages would have been repeatedly sampled. To avoid confusion, we have omitted the word ‘data-point’ throughout the revised manuscript and refer to prevalence surveys. See also **Reply by Authors to 2.3.1**, where we have clarified that all surveys were conducted at village level.

2.3.6 Spatial unit: Line 203. I was unclear on the spatial unit of analysis here. You describe having 571 locations but then say the unit is health districts in Cameroon. It would be helpful for the readers not familiar with Cameroon to be given an idea of the size of a health district to understand the spatial resolution of your analyses/limitations of assumed location. Seems from what I’ve found online that the country is broken up into 189 health districts which doesn’t fit with your locations. Please clarify and explicitly explain any assumptions/approximations/aggregations for the location used in the analysis.

Reply by Authors to 2.3.6: We thank the referee for pointing this out. We now state that we used village-level surveys:

“We used village-level surveys (our spatial unit). The years of data collation were grouped according to whether the village-level data had been collected prior to CDTI (pre-control) or following its implementation.”

2.3.7 Covariate selection: Lines 217-219. This is not clearly described. I would suggest changing the order of this section and explain how variables were selected with GLMs before moving to fitting the full binomial geostatistical model (I would also suggest specifying that this is the model that you fit rather than just the PrevMap function). I also saw the use of the term ‘spatial GLM’ in your results and wanted to clarify that here you are just talking about a GLM for model selection rather than a geostatistical model?

Reply by Authors to 2.3.7: We are grateful to the reviewer for indicating that our description of the process was not entirely clear to the reader. We have re-written the sub-section of the Methods describing the geostatistical model as follows:

"In order to model the spatial variation of microfilarial prevalence, a MBG approach was used to take into account the spatial correlation between observations and the effects of environmental covariates [41,42]. We followed procedures previously described [28,41]. Firstly, we used logistic regression—a type of generalized linear model (GLM) for binary dependent variables—with a stepwise method

based on minimising the Akaike Information Criterion (AIC), to identify statistically significant environmental covariates, setting the significance level at 0.05. Multicollinearity checks were carried out using analysis of variance inflation factors (VIF) [43]. Predictors with VIF greater than 10 were removed (Supporting Information File, S2 Text). Secondly, from the residuals of these binomial GLM models, semi-variograms were prepared using the Matérn correlation function. Finally, the covariates retained were included in the spatial binomial logistic regression model, implemented using the binomial.logistic.MCML function of the PrevMAP R package version 1.5.4 [44], which estimates model parameters using Monte Carlo maximum likelihood (MCML) [45,46]. This process was repeated until convergence was reached and for all different time periods. In the first instance, the model was fitted to the overall, 1971–2020 interval to assess the effect of intervention period. Subsequently, the model was fitted separately to the baseline (1971–2000), 2001–2010 and 2011–2020 periods. All analyses were performed with R software version 4.1.0 [47]. Full details of the MBG approach and parameter estimation procedures are provided in Supporting Information File, S2 Text, which also describes the rationale for spatial prediction and, of particular interest for detection of hotspots, the calculation of exceedance probability, which we used to prepare predictive maps that microfilarial prevalence is at least 35%. The resolution of the output maps is 5 km x 5km."

2.3.8 Variable selection: It also wasn't clear to me how you selected variables – did you fit a single GLM with all the variables in it and then just select those that had p<0.05? A better approach would be assessing goodness of fit e.g. with AIC or cross-validation.

Reply by Authors to 2.3.8: As described in **Reply by Authors to 2.3.7**, we used a stepwise method using AIC for covariate selection.

2.3.9 I would also move 'The degree of significance for each variable was set at 5%' (lines 220-221) before the multicollinearity sentence so it's clear what you define as being 'statistically significant'.

Reply by Authors to 2.3.9: This has been done following the reviewer's suggestion. See **Reply by Authors to 2.3.7**.

2.3.10 It would also be helpful to know how this process took place considering your data was subdivided into different periods i.e. whether you conducted covariate selection using all the data together or to define different models for the different periods. Seems from results like you fit independent geostatistical models for each period and then one model to all of the data with period as a covariate, but it would also be helpful to explain this process clearly to the reader.

Reply by Authors to 2.3.10: See also **Reply by Authors to 1.6.2:**

"In the first instance, the model was fitted to the overall, 1971–2020 interval to assess the effect of intervention period. Subsequently, the model was fitted separately to the baseline (1971–2000), 2001–2010 and 2011–2020 periods".

2.3.11 Multicollinearity checks: Lines 219-220: Please give the details of your multicollinearity checks i.e. what VIF cut-off did you use.

Reply by Authors to 2.3.11: The details of the multicollinearity checks had been provided in the SI File, now Supporting Information File Text, S2. In the revised text, we have sign-posted the reader to the SI File: "Multicollinearity checks were carried out using analysis of variance inflation factors (VIF)

[43]. Predictors with VIF greater than 10 were removed (Supporting Information File, S2 Text)", where we state:

"For each GLM, only statistically significant variables were kept. Multicollinearity checks were carried out using analysis of variance inflation factors (VIF) [10,11],

$$VIF_j = \frac{1}{1 - R_j^2} \quad \text{(S5)}$$

where R_j^2 is the coefficient of determination of the jth predictor when the latter is explained by the other predictor variables [10]. Predictors with VIF greater than 10 were removed from the model using a backward procedure. The GLM models retained contained only significant predictors whose VIFs were less than 10."

2.3.12 Nugget effect – it seems like this wasn't included in your models, but there is evidence of a nugget in your variogram C.

Reply by Authors to 2.3.12: We did estimate the (relative) nugget and we now provide the values in the caption of Fig 3. The nugget for variograms A and B was zero. The nugget for variogram C was 1.79.

2.3.13 Correlation function: Presumably you assumed an exponential correlation function, but would be good to specify this in the main text.

Reply by Authors to 2.3.13: We have now stated in the Methods section of the Main Text that we used a Matérn correlation function (see **Reply by Authors to 2.3.7**). For the benefit of the reviewer, the correlation function is given in Eqn. (S2) of the revised S2 Text of the Supporting Information file.

2.3.14 Prediction: No mention of this in your methods. Presumably these were marginal predictions?

Reply by Authors to 2.3.14: We have now re-structured S2 Text of the Supporting Information file to provide details of the approach for spatial prediction; see Eqn. (S7).

2.4 Results: Results has all the expected results in it but some tidying up of figures and text is required to make this section clearer and more specific. Specific comments:

2.4.1 Visualisations

Scale bar in Figures 1 and 2: Would be nice to have a scale bar on your map.

Prevalence scales in Figures 1 and 2: The prevalence scales aren't very clear - the use of '00' and the way intervals are shown. The use of different prevalence scales used between the two figures also makes it challenging to compare the changes over time. I would use the same scale across both figures.

Datapoints: Making your datapoints slightly transparent would help visualising overlap of datapoints.

Size of Figure 2: The size makes this challenging to see the points clearly and I would remove the region names as they're too small to read.

Figure 4 and text lines 299-305: Please add a legend key to show prevalence and SE.

Caption of Figure 4 needs to be more specific e.g. Mean predicted prevalence from the period-specific binomial geostatistical models. Please also make this clearer in the text.

Different colours are used for prevalence levels for A, C and E. These need to be the same to allow for comparison between the figures.

Exceedance probability: Great to see these.

Figure 5: check your prevalence scales, they have different colours in panel C which makes it hard to compare across them.

Reply by Authors to 2.4.1: We have now added a scale bar in Fig 1 and Fig 2. We have also replotted the figures with the same microfilarial prevalence scales to facilitate comparison. We have added text to explain this in the Methods section of the Main Text and the caption of Fig 1 and Fig 2 (see also **Reply by Authors to 2.3.4**). We will not be able to make the markers in these figures more transparent. In Fig 1 we have made the names of the regions slightly larger. As we refer to the regions by name in the Discussion, we would prefer not to omit these names. We have modified the caption of Fig 4 as suggested, added 'Predicted prevalence' and 'Standard error (SE)', and have re-done the figures to keep the colours the same in panels A, C, and E as indicated. For the exceedance probability, we have included explanatory text in S2 Text of the Supporting Information file, and have replotted Fig 5 to keep the colours the same in all three panels, as pointed out.

2.4.2 Use of terminology

'spatial GLM' (Line 273): It would be clearer to call this a binomial geostatistical model. I see the term GLM is also used in line 285, but the supplementary tables seem to refer to geostatistical models.

Table 1: It would be helpful to be more specific with your terminology here, e.g. give it a title 'Parameter estimates for the binomial geostatistical model for microfilarial prevalence for the complete period 1971-2020….'

Please include confidence intervals, more interpretable than SE and z-values. Please also include for phi and sigma^2.

I'm not really clear on the significance of this 'all period' model – it doesn't get used in the predictions, as the analysis seems to be really conducted using period-specific models. This all needs to be much clearer in the methods section.

Line 312: This is mean predicted microfilarial prevalence. Please be careful with the terminology used.

Line 324: typo for 'predictive'.

Reply by Authors to 2.4.2: The terminology has been clarified as suggested both in the text and the tables (of Main Text and Supporting Information); 95% confidence limits are now provided rather than z- and SE values. The purpose of fitting to the whole study period has been given in the Methods (see also **Reply by Authors to 1.6.2** and to **2.3.7**). Mean predicted microfilarial prevalence has been used. The typo for 'predictive' has been corrected.

2.5 Discussion: I found this a long and quite challenging section to read/digest and would suggest some substantial cutting and restructuring of the discussion section to more clearly communicate the key points that the author wishes to make. There is a lot of information but it's often delivered in quite an unstructured way without linking it to previous sentences or reflecting on it. I suggest starting each paragraph with a sentence that articulates the main point you wish to make, followed by sentences describing the evidence, giving references to literature, reflections etc.

Generally, I don't feel that the first half of discussion did justice to the findings in the results. Much of it before line 400 reads like a results section and is hard to follow, but it is much better after this point (with lots of interesting discussion points).

From the very beginning of the discussion I would expect a much clearer discussion of i) spatio-temporal variation in predicted prevalence, comparison with other study findings and considerations of changes in the disease's epidemiology and data collection needs; ii) exceedance probability and implications for disease control; iii) considerations of data/model fit/other limitations. I suggest cutting the discussion down significantly (particularly the first half, much of which I think isn't needed) and restructuring paragraphs so the reader can clearly identify the main takeaway points.

Specific comments:

2.5.1 Paragraph 1:

This reads a bit too much like the methods. I think helpful to give a quick overview of your analysis, but I would suggest 2 sentences explaining the study aims/data followed by a very top-level overview of the main results from the results.

Line 338: 'predicted prevalence' not just 'prevalence'

Line 339: 'inedited data' - typo?

Line 340: 'to be presented elsewhere' – unclear what this means?

Line 342: Unclear what the relevance of the comparison to reference 34 is.

Reply by Authors to 2.5.1: We have re-ordered the Discussion. We have omitted details that pertained to the Methods (see also **Reply by Authors to 1.7.1** and to **1.7.2**).

We have used 'predicted prevalence' and not just 'prevalence' where appropriate.

The word 'inedited' does no longer appear in the revised text. For the benefit of the reviewer, this was not a typo. "Inedited" refers to something unpublished or not edited. It comes from the Latin word inēditus, which means "not made known".

The clause 'to be presented elsewhere' does no longer appear in the revised text.

The comparison with Ref. [34] is relevant, as we are indicating that we identified more prevalence surveys than this reference for a comparable study interval.

2.5.2 Paragraph 2:

Given this study was a prediction study rather than a causal inference study I would leave any interpretation of coefficients for later on in the discussion (or preferably not at all).

I would make your second paragraph the one that discusses your most important results which are your findings about the spatial distribution of predicted prevalence, how that changes over time.

Reply by Authors to 2.5.2: We have moved the interpretation of coefficients to the SI File. In the revised text we say:

"Although our study is a prediction rather than a causal inference study, we offer a cautious interpretation of the environmental coefficients in Supplementary S3 Text".

2.5.3 Paragraph 3:

It was not clear to me what the point of this paragraph was, there didn't seem to be any actual comparison but rather just a description of what other modelling has been done.

Reply by Authors to 2.5.3: This description provides an important backdrop for comparison with our results.

2.5.4 Paragraph 4:

This wasn't very clear to me. Again, a first sentence that explains the main point of the paragraph would be helpful and would help you to focus the paragraph more clearly. E.g. 'Our prevalence predictions were similar to findings of a recent study.'

Check your referencing style for including in text citations of papers – I would expect these to include author names rather than just 'in [33] and [34]'.

Final sentence of the paragraph is not linked to the rest of the text at all.

Reply by Authors to 2.5.4: The paragraphs have been revised. The re-structured Discussion comprises the following sub-headings (see also **Reply by Authors to 1.7.2**):

- MBG mapping of onchocerciasis in Cameroon
- Implications of our findings for onchocerciasis elimination in Cameroon
- Limitations
- Future research avenues

Regarding referencing style, we have added first author surname et al. followed by the numbered reference where appropriate.

2.5.5 Limitations:

This was better. Remember that how well your prevalence surveys captured transmission for your models is dependent on how stable transmission was during the study periods.

Lines 403-404: how is the MBG framework designed to overcome the limitations of data not collected for mapping and temporal trends?

Lines 417-425: Why wasn't this variable included?

Reply by Authors to 2.5.5: We have included the comments by the reviewer in the Limitations section. We have omitted text in Lines 403-404 (of the original submission). Regarding distance to rivers, as the layers of environmental covariates were obtained from the NASA portal, this variable was not included, but we have acknowledged this in the Limitations section.
