# Supplementary material for: Model-Based Geostatistical Mapping of the Prevalence of Onchocerca volvulus in Cameroon between 1971 and 2020
Source: PLoS Negl Trop Dis. 2025 Mar 28;19(3):e0012250. doi: 10.1371/journal.pntd.0012250 (PMC11978065; doi:10.1371/journal.pntd.0012250)
Supplement: Attachment — Submitted filename: Reviewrs report - PLoS NTD July 2024.docx [file pntd.0012250.s002.docx]

**Title:** **Model-based geostatistical mapping of the prevalence of *Onchocerca volvulus* in Cameroon between 1971 and 2020**

**General comment**: This is a well-written manuscript and an important piece of work that provides geostatistical modeling predictions of onchocerciasis prevalence in Cameroon from 1971 to 2020. It offers further evidence of the effectiveness of ivermectin in controlling and eliminating onchocerciasis. The data output from this work has the potential to be applied to transmission models to inform future trends and predictions toward onchocerciasis elimination.

**Abstract:** Well-written. It is clear, concise, and contains all the relevant details.

Introduction: Well-written. It provides a good historical background on interventions against onchocerciasis. However, since this work focuses on building geostatistical models for onchocerciasis in Cameroon, and not on the transmission modeling use case of geostatistical modeling outputs (Lines 172-175), it is recommended to exclude the latter from the introduction. Use cases for geostatistical modeling outputs can be provided in the Discussion section, as they are out of place in the introduction.

**Methods:**

Data – More information is needed in this section to understand what onchocerciasis data was used to fit the models. For example, please provide the numbers and distribution of data across all data sources. How much of the data was obtained from the literature, the ESPEN portal, etc.? For what years? Were there any data duplicates? For instance, did ESPEN’s report of prevalence rates for a specific area differ from rates reported for a similar area in data obtained from the literature? If there are examples of such instances, how did the authors handle them? I see that this sections links to a protocol cited in ref 39, but it is good to provide adequate information, so readers don’t have to go elsewhere to get a complete understanding of the study.

Onchocerciasis endemicity is not driven solely by climatic factors. Not including key socio-demographic variables as covariates in this model could be considered a significant omission. The authors should justify why they have chosen not to account for any socio-demographic covariates in this work.

**Statistical analysis:**

Conversion of nodule to microfilaria prevalence and its stratification – The terms pre-intervention, baseline, and pre-control (Lines 194-196) are used interchangeably. Although they all mean the same thing, it would be better to be consistent and use one term throughout the manuscript.

Geostatistical model – It is unclear if a separate model was fitted to each of the year bands and time periods described in previous sections. From the empirical semi-variograms in Fig. 3, it appears that each time period was modeled separately. Please clarify this in this section.

**Discussion:**

The first paragraph in the Discussion section goes into more description of the data. This may be better suited in the Methods section, as it seems out of place to start the Discussion with this information.

To improve flow and readability, I would also recommend re-ordering the Discussion as follows;

- Implications of our findings for onchocerciasis elimination in Cameroon
- MBG mapping of onchocerciasis in Cameroon
- Limitations
- Future research avenues

Use of the word ‘significant’ – The authors should use ‘statistically significant’ if that is what they intend to convey
